# SpinachDB: A Well-Characterized Genomic Database for Gene Family Classification and SNP Information of Spinach

**DOI:** 10.1371/journal.pone.0152706

**Published:** 2016-05-05

**Authors:** Xue-Dong Yang, Hua-Wei Tan, Wei-Min Zhu

**Affiliations:** 1 The Protected Horticulture Institute, Shanghai Academy of Agricultural Sciences, Shanghai, China; 2 College of Horticulture, Nanjing Agricultural University, Nanjing, Jiangsu, China; Youngstown State University, UNITED STATES

## Abstract

Spinach (*Spinacia oleracea* L.), which originated in central and western Asia, belongs to the family Amaranthaceae. Spinach is one of most important leafy vegetables with a high nutritional value as well as being a perfect research material for plant sex chromosome models. As the completion of genome assembly and gene prediction of spinach, we developed SpinachDB (http://222.73.98.124/spinachdb) to store, annotate, mine and analyze genomics and genetics datasets efficiently. In this study, all of 21702 spinach genes were annotated. A total of 15741 spinach genes were catalogued into 4351 families, including identification of a substantial number of transcription factors. To construct a high-density genetic map, a total of 131592 SSRs and 1125743 potential SNPs located in 548801 loci of spinach genome were identified in 11 cultivated and wild spinach cultivars. The expression profiles were also performed with RNA-seq data using the FPKM method, which could be used to compare the genes. Paralogs in spinach and the orthologous genes in *Arabidopsis*, grape, sugar beet and rice were identified for comparative genome analysis. Finally, the SpinachDB website contains seven main sections, including the homepage; the GBrowse map that integrates genome, genes, SSR and SNP marker information; the Blast alignment service; the gene family classification search tool; the orthologous and paralogous gene pairs search tool; and the download and useful contact information. SpinachDB will be continually expanded to include newly generated robust genomics and genetics data sets along with the associated data mining and analysis tools.

## Introduction

Spinach (*Spinacia oleracea* L.), which originated in central and western Asia, belongs to the family Amaranthaceae. Spinach is particularly resistant to cold, has wide adaptability, and is cultivated worldwide, mostly in temperate regions. As one of most important leafy vegetables, spinach has a high nutritional value, and is rich in carotene, vitamin C, amino acids and iron, among others [1]. Recent studies show that spinach may help to protect people against inflammatory problems, oxidative stress-related problems, cardiovascular problems, and cancers [2–4]. In 2013, the total harvested area of spinach reached 910833 Ha and gross production value (USD current) reached $11.995 billion worldwide, according to FAOSTAT statistics (http://faostat3.fao.org).

Because spinach is a particularly desirable vegetable, germplasm resources and breeding are important. At present, spinach cultivars in the USA, Europe and Japan, are mostly F1-hybids. Molecular breeding for complex traits in crop plants requires understanding and manipulation of many genes. Utilization of genome-wide molecular markers is an effective tool for plant breeding, which drives researchers to focus on constructing detailed genetic maps with high density markers [5]. A genetic map of spinach was constructed using 101 AFLP and 9 microsatellite markers, and the result showed a small chromosomal region co-segregating with sex determination in the species [6]. In the recent years, 10 AFLP and 2 male-specific markers were identified in close vicinity to the X/Y locus and a single major gene responsible for the monoecious condition was found [7]. Spinach is a model for genetic and physiological studies on sex determination and expression, and the molecular basis of sex determination needs further study [7]. Study on the spinach C class floral identity genes indicated that gene SpAGAMOUS was differentially expressed prior to reproductive organ development and involved in the sexual dimorphism of spinach [8].

In recent years, with the advent of high-throughput sequencing technologies, genomic nucleotide sequence data of many crops has accumulated, and *de novo* assembly of genomic sequence data can provide whole-genome sequences, which are valuable resources for investigating the genetic characteristics; identification of the candidate genes especially associated with agronomic traits; and understanding of important evolutionary processes [9]. With the accumulation of the large amount of plant genome sequences, various genomics databases have been constructed for such species, including genome database for carrot [10], rice [11], radish [12] and gene family database like SALAD database [13], GreenPhylDB [14] and Phytozome [15].

Spinach possesses a small genome, thus is suitable for basic genomic studies, and many physiologically important genes have been cloned from the species [16]. Recently, the spinach genome has been sequenced and *de novo* assembled [17,18]. The genome sequence and gene predictions are available at The *Beta vulgaris* Resource website (http://bvseq.molgen.mpg.de) [18]. Although researchers can browse genes and make a blast query in that website, useful tools for deeply mining spinach genes are still lacking. Thus, it is urgent to construct a central database for spinach to efficiently store, annotate, mine and analyze the genomics and genetics datasets. Therefore, we developed SpinachDB, to store assembled and annotated EST transcripts, predicted metabolic pathways, and EST-derived simple sequence repeat (SSR) and single nucleotide polymorphism (SNP) markers. A set of tools and user-friendly query interfaces have also been developed in the database to help researchers in identifying and deciphering biologically important information from the datasets. SpinachDB will be continually expanded to include newly generated robust genomics and genetics data sets and the associated data mining and analysis tools.

## Materials and Methods

**Fig 1:**
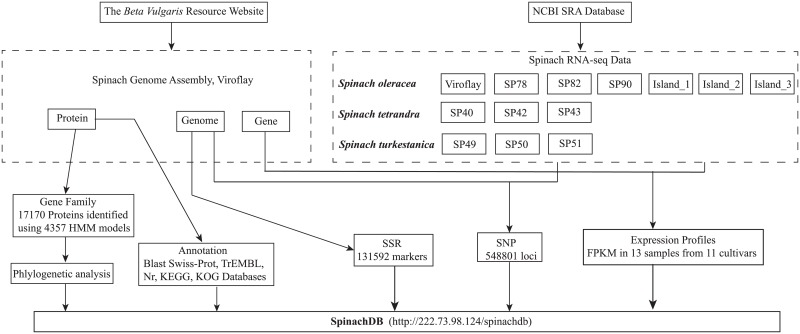
The workflow for data mining and construction of SpinachDB.

The workflow was, essentially, gene annotation, SSR and SNP detection, and expression profiling of genes using spinach genomic and transcriptomic data. This was followed by pooling the data into a database and providing the researcher with a user-friendly website interface (Fig 1).

### Collection and annotation of genome resources

Since the spinach genomic data was available in The *Beta vulgaris* Resource website (http://bvseq.molgen.mpg.de/Genome/Download/Spinach/), we downloaded the Spinach genome assembly version 1.0.1 and annotation version SpiSet-1 (August 2014) for further analysis. RNA-seq raw data were also downloaded in the NCBI SRA (Sequence Read Archive) database (http://www.ncbi.nlm.nih.gov/sra) [19]. All of the spinach proteins were annotated by search, using NR (Non-reduntant, NCBI), Swiss-Prot and TrEMBL (www.uniprot.org) [20], KEGG (http://www.genome.jp/kegg) [21], and KOG (http://www.ncbi.nlm.nih.gov/COG) [22] databases using BLASTP with a cut-off E-value of 10^−5^. SSRs were identified using the MIcroSAtellite (MISA) identification tool (http://pgrc.ipk-gatersleben.de/misa/) based all the spinach genome sequences. For mono-, di-, tri-, tetra-, penta- or hexanucleotide SSRs, the minimum number of units were set to be 10, 6, 5, 5, 5 and 5 repeats, respectively. The software Primer3 (http://bioinfo.ut.ee/primer3/) was used to design potential primers for each SSR, with default parameters following the previous rules [23].

### Classification of gene families

The HMM (Hidden Markov Models) models of gene families were downloaded from the Pfam database (http://pfam.xfam.org) on September 26, 2015 [24]. The proteins of another 12 representative species in the plant kingdom including algae, moss, fern, spruce, *Arabidopsis*, rice, grape, poplar, tomato, apple, soybean and sugar beet were also downloaded. The proteins of *Arabidopsis* and sugar beet were downloaded from TAIR 10 (http://www.arabidopsis.org) and The *Beta vulgaris* Resource website respectively; the others were downloaded from the Phytozome portal (http://phytozome.jgi.doe.gov, v10) [15]. For those genes that had several corresponding protein products due to splicing models, only the primary ones were retained for further analysis. The hmmsearch program (http://www.hmmer.org, v3.1b1) [25] was used to identify gene families with the same E-value of e^-5^. The output of all identified gene families was classified according to the species. The software orthoMCL (http://orthomcl.org/orthomcl, v2.0.3) [26] was performed to identify orthologous and paralogous gene pairs in *Arabidopsis*, grape, rice, sugar beet and spinach.

### Expression profiles of genes and SNP mining

Thirteen SRA files were used to characterize the gene expression profiles and for SNP mining of spinach, including 7 from cultivated *S*. *oleracea* (Viroflay cultivar, SRR1542623; SP78 cultivar, SRR1766310; SP82 cultivar, SRR1766311; SP90 cultivar, SRR1766312; Island cultivar, SRR1763297, SRR1763298, SRR1763299), 3 from *S*. *tetrandra* (SP40 cultivar, SRR1766329; SP42 cultivar, SRR1766329; SP43 cultivar, SRR1766329) and 3 from *S*. *turkestania* (SP49 cultivar, SRR1766332; SP50 cultivar, SRR1766333; SP51 cultivar, SRR1766334). The fastq-dump program in the SRA Toolkit was used to release fastq format files from SRA files; then NGS QC Toolkit (http://www.nipgr.res.in/ngsqctoolkit.html, v2.3.3) was used to obtain high quality reads which contained at least 90% bases beyond Phred-Qual 20 [27]. The program Tophat (https://ccb.jhu.edu/software/tophat/index.shtml, v2.1.0) [28] was used to map the reads to the spinach genome; then the expression profile of all spinach genes was obtained with FPKM (Fragments Per Kilobase of exon per million fragments Mapped) value using software Cufflinks (http://cole-trapnell-lab.github.io/cufflinks, v2.2.1) that under the guidance of annotated gene models with a GFF file [29,30]. Meanwhile, the mapped profiles were used to identify the different bases while being compared with reference spinach genome sequence. The Genome Analysis Toolkit (GATK) software package (http://www.broadinstitute.org/gatk/, v3.5) [31] was used for SNP calling using HaplotpeCaller with default parameter before hard filters were applied to the call sets. Another python script in software Seqgene (http://sourceforge.net/projects/seqgene/, v2.5) [32] were used to identify SNPs in consideration of the quality of bases, the minimum coverage and percentage of SNP allele calling.

### Server and website construction

Linux system CentOS6.6 (http://www.centos.org) was installed on an HP blade server machine with 80 threads and 700 GB memory. The MySQL database server (http://www.mysql.com, v5.1.73) was installed for storing all genomic and website data. The Apache web server with PHP (v5.3.3) was used for powerful website service, and the software Joomla (http://www.joomla.org, v2.5) was used for user-friendly website interface construction. Some frequently used bioinformatics programs like GBrowse (http://gmod.org, v1.70) and wwwblast (ftp://ftp.ncbi.nlm.nih.gov/blast/executables, v2.2.26) were installed to provide user-friendly search capability for genes of interest. Some Perl and CGI (Common Gateway Interface) scripts were built within to provide additional search services.

## Results and Discussion

### Overview of the database contents and functions

**Fig 2:**
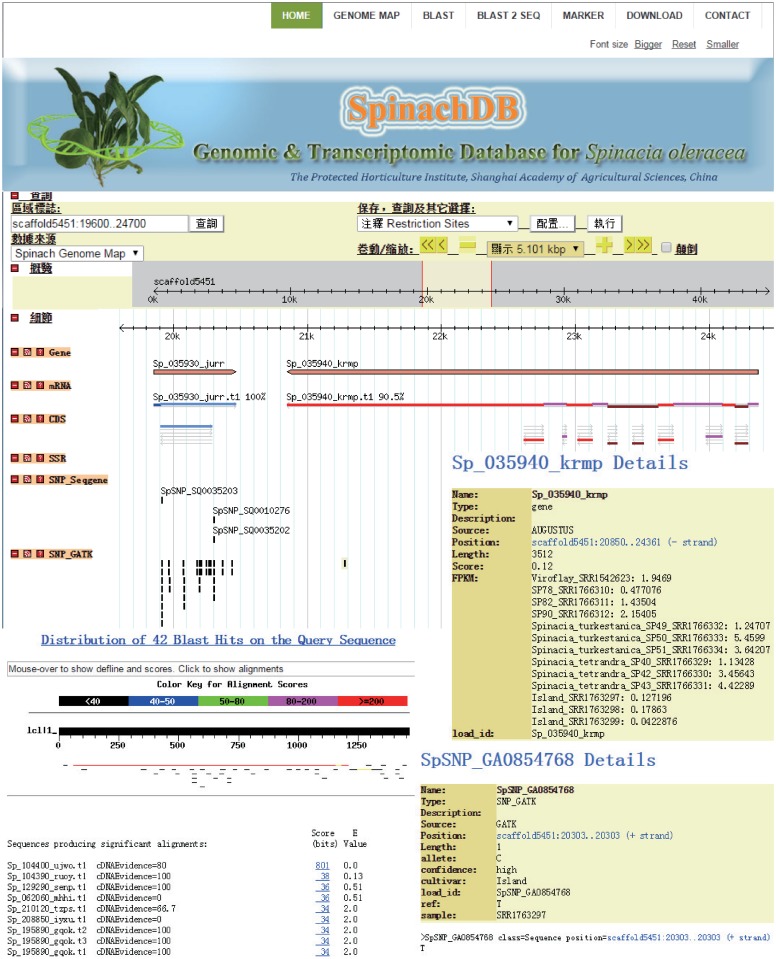
The organizational structure of the SpinachDB website.

The genomic and transcriptomic data of spinach are publicly available in SpinachDB (http://222.73.98.124/spinachdb). The Fig 2 shows that the website contains seven main sections, including the homepage; the GBrowse map that integrates genome, genes, SSR and SNP markers information; the Blast alignment service; the gene family classification search tool; the orthologous and paralogous gene pairs search tool; and the download and useful contact information.

### The genome of spinach and SSR identification

The span of the updated genome assembly of spinach was 489 Mb in total, with 21702 predicted genes with high confidence found in the *Beta vulgaris* Resource website. Based on the information in that website, the software Augustus (http://augustus.gobics.de/) predicted 40309 genes, among which only 21702 were high-evident ones that were collected for the dataset used for final gene assembly. To functionally categorize these genes, there were 15550, 18864, 18659, 7956, 18634 genes assigned annotation after aligning with Swiss-Prot, TrEMBL, KEGG, KOG, NR and NT databases, respectively (Table A in S1 File). These annotations give insight into spinach genes, based on molecular research on corresponding genes of other organisms.

A total of 131592 SSRs were found in the whole-genome sequence of spinach cultivar Viroflay, including 94741, 16749, 15379, 1748, 1754 and 1221 for mono-, di-, tri-, tetra-, penta- and hexanucleotide repeats, respectively. The number of genomic SSRs in spinach was almost 2.7 times that in carrot (48398 SSRs) while the genome sizes of these two species were similar. A collection of 13229 SSRs that fit the definition of a compound SSR were found in this study. Among all of the SSRs, the A_10_ and T_10_ type were the most abundant ones. In addition, to develop SSR markers useful for experimental validation, 57519 pairs of primers specific for SSRs were designed using Primer3 software. The SSRs and corresponding primers were integrated in the GBrowse tool, so as to provide useful information for genetic map construction.

### Gene expression in different samples

Recently, biology has become a data intensive science because of huge data sets produced by high throughput molecular biological experiments in the field of genomics, transcriptomics, proteomics, and metabolomics. These data is useful for constructing molecular biological networks [33]. To characterize the expression profiles of genes in different cultivars, the RNA-seq data of four spinach cultivars that had been sequenced in two stand-alone projects were collected from the NCBI SRA database. To reduce false positivity and bias, the low-quality bases were filtered using the software NGS QC Toolkit (http://www.nipgr.res.in/ngsqctoolkit.html) [31]. After that, 10.57 million, 1.04 million, 0.88 million, 1.15 million, 0.87 million, 1.48 million, 1.60 million, 1.03 million, 0.97 million, 0.85 million, 46.08 million, 43.61 million and 47.06 million sequencing bases, for cultivars Viroflay, SP40, SP42, SP50, SP51, SP78, SP82, SP90 and three samples of cultivar Island respectively, were retained for further analysis. The assembly of transcriptomic sequences from these spinach cultivars was performed using Tophat and Cufflinks with a guided GFF (General Feature Format) file, yielding expression profiles for each gene and the corresponding isoforms. There were 19715 (90.8%), 19639 (90.5%), 19326 (89.1%), 19559 (90.1%), 19508 (89.9%), 19653 (90.6%) and 20894 (96.3%) genes expressed in cultivated cultivars Island 1, Island 2, Island 3, SP78, SP82, SP90 and Viroflay, respectively (Table B in S1 File). For the wild spinach, there were 19646, 16599, 17117, 17927, 19984 and 20014 genes expressed in sample SP40, SP42, SP43, SP49, SP50 and SP51 (Table C in S1 File).

### SNP detection in different cultivars

SNP detection is usually performed by mapping the DNA sequencing reads to the referent genome sequences with pipelines and software, such as GATK (https://www.broadinstitute.org/gatk/) and SOAPsnp (http://soap.genomics.org.cn/). Although DNA-oriented sequencing reads were regularly used for SNP prediction for better results, RNA-oriented sequencing reads such as RNA-seq have also been performed occasionally, but increasingly, in many studies. In some studies, the RNA-seq data and EST sequences were also used to predict potential SNPs. The subsequent SNP validation experiment supported the use of RNA-seq and also EST data for contribution to SNP identification. The validation of selected high-confident SNPs in tea plant [34], longan [35], and pineapple [36] were carried out with nearly half of them having been validated.

Although there were several studies on the SNP and comparative analysis using transcriptomic data, they were conducted by mapping reads to assembled expressed transcripts rather than whole genome sequencing [37,38]. In this study, a total of 1125743 potential SNPs located in 548801 loci of spinach genome were identified. Initially, we attempted to use the software packages SOAP and SOAPsnp to detect SNPs, but too much false positive results were obtained. The professional software GATK called 1074400 potential SNPs in 535439 loci. Among them, 169915 (15.8%) were defined as high confident SNPs that passed hard filters, whereas 834549 were intermediate confident SNPs and 69936 Low quality SNPs. The cultivar SP42 and SP43 of *Spinacia tetrandra* contained more SNPs than other cultivated or wild spinach, which indicated its relationship with other cultivars were more far away. In addition, we used the software Seqgene with lower criteria, resulting in a total of 51343 SNPs in 33631 loci.

The differentiation of gene expression and SNPs among these cultivars was a useful resource for molecular biology as well as for breeding. Some of these differentiated genes could be related to resistant/tolerant genes or to those for high nutritional value. The SNPs of interest in gene regions could be easily selected for validation according to gene annotation. At the pre-breeding stage, the validated SNPs were useful in selecting good breeding materials and good F1 hybrid lines, which could potentially facilitate the breeding of cultivars that are fleshy, high yielding, highly tolerant to abiotic stresses and highly resistant to pests.

### Orthologous and paralogous genes

**Fig 3:**
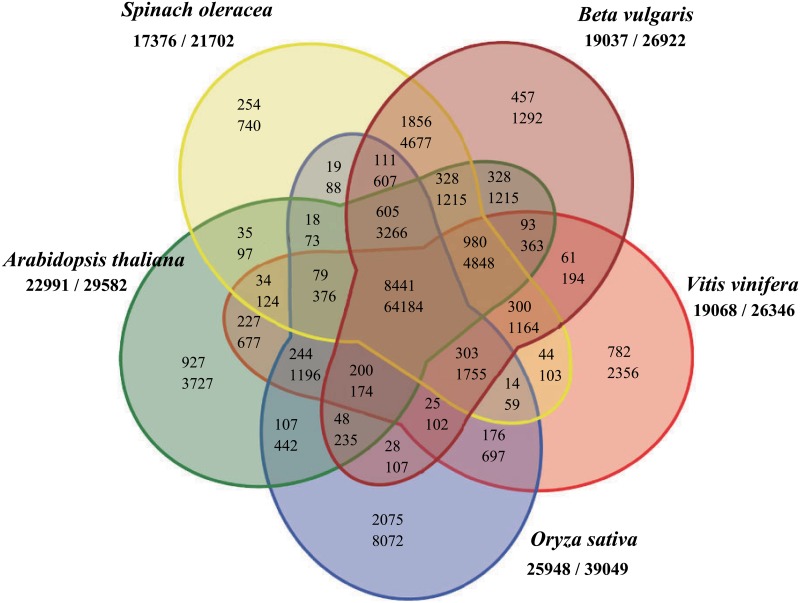
The Venn diagram shows shared and distinct cluster classes from an orthoMCL analysis of proteins from Arabidopsis thaliana, Oryza sativa spp. indica, Vitis vinifera, Beta vulgaris and Spinach oleracea. Numbers below each species name indicate numbers of clustered genes and of all genes in the corresponding genome. For each area in the Venn diagram, the top line and the bottom line represent the number of orthoMCL clusters and the number of all accumulated shared genes in all organisms.

The concepts of orthology and paralogy originated in 1970s, and have been used for functional annotation and classifications on large scale whole-genome comparisons [39]. To give insight into the crossing-referencing and classification of genes from multiple species among the flowing plants, the well-studied plants *Arabidopsis*, rice, and grape from different clades; sugar beet in the same family; and spinach were selected to detect orthologs and paralogs. Among these species, the percentage of classified genes was fairly high, i.e. between 66.4% (rice) and 83.9% (*Arabidopsis*). All five of these species were flowering plants and the orthology they shared consisted of 64184 orthologous gene pairs in 8441 groups. The four eudicots shared more orthologous genes, while rice, the monocotyledonous species, shared the fewest orthologs with the others. The distribution of orthologous gene pairs was totally consistent with the genetic relationship. The species in the same family shared the largest number of orthologous gene pairs, and then with other eudicots, and the least with the monocot (Fig 3, Table C in S1 File).

**Fig 4:**
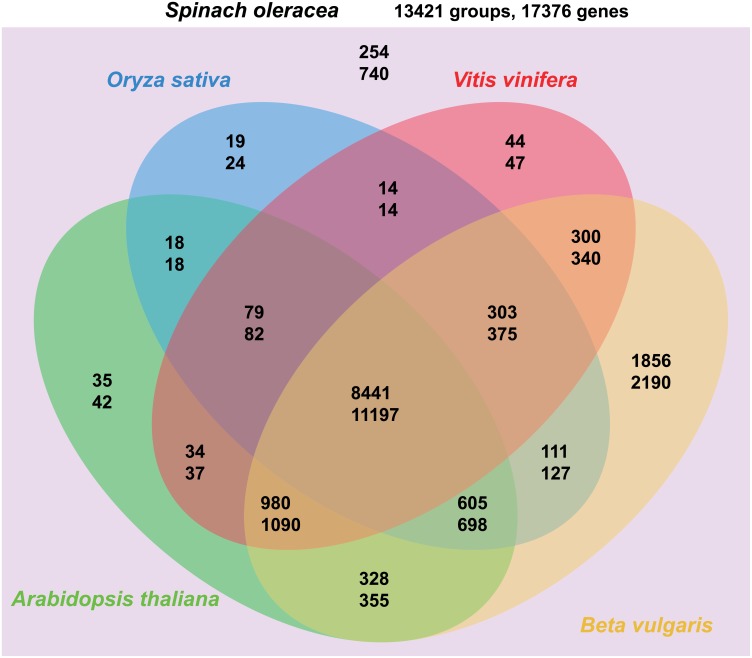
The Venn diagram shows the number of clustered groups and genes that spinach shared with other four plants. For example, the numbers 328 and 355 at the bottom of this figure indicate that for all clustered groups of Arabidopsis thaliana, Beta vulgaris and Spinach oleracea, the number of these groups is 328 and there are 355 spinach genes in these groups.

To investigate the orthologous genes in spinach, a Venn diagram was drawn to show the number of clustered groups and genes that spinach shared with the other four plants (Fig 4). A total of 17376 genes were identified in 13421 groups in this study. Among them, 740 spinach genes in 254 groups were identified to be paralogous genes, without corresponding orthologous genes in the other four species. A total of 11197 spinach genes were found to be orthologs that were shared with all other four species. Another 1090 spinach genes were orthologous with sugar beet, *Arabidopsis* and grape simultaneously, in 980 groups. There were 2190, 355, 47 and 24 spinach genes that were found only to have orthologous genes with sugar beet, *Arabidopsis*, grape and rice, respectively. These orthologous and paralogous gene pairs can be usefully employed to facilitate both evolutionary and functional analyses of spinach.

### Gene families

A total of 16230 HMM models from Pfam database were used to search proteins in 13 organisms. A total of 301184 proteins were catalogued to 5699 HMM models. Among all 21702 proteins of spinach, 15741 proteins were catalogued into 4351 HMM models (Table D in S1 File). Among these 13 organisms, the spinach proteins had the largest number of proteins in 272 HMM model groups, such as the gene families FeoB_N (PF02421), MS_channel (PF00924), MTS (PF05175), PD40 (PF07676) and Whirly (PF08536). Users, especially molecular researchers without a robust programming background, can easily find the name and sequence of proteins in a particular gene family and perform a genome-wide analysis of the gene family in a spinach study.

**Fig 5:**
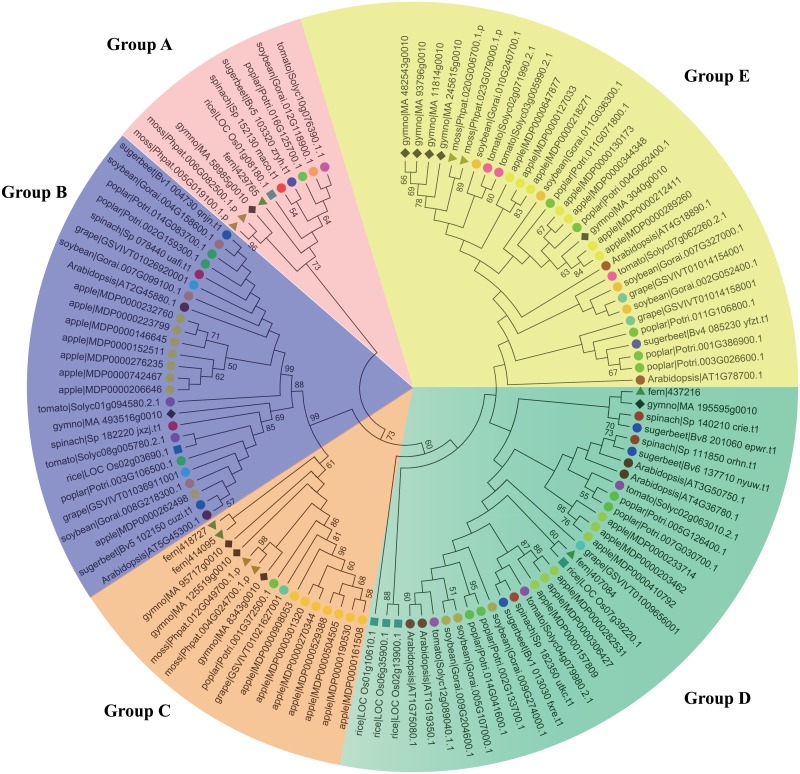
Phylogenetic tree of twelve plants constructed based on protein sequences of BES1/ domains using the maximum-parsimony method. The different species can be distinguished by different shapes and colors.

In order to explain a possible usage for these classified gene families, the genome-wide identification and analysis of BES1/BZR1 in spinach were performed. The BES1 gene family is a class of plant-specific transcription factors that play a key role in the Brassinosteroid signaling pathway. The BES1 gene interacts with the basic helix-loop-helix protein BIM1 to synergistically bind to E box (CANNTG) sequences present in many BR-induced promoters [40]. In this study, it was straightforward to obtain the names and sequences of 118 proteins from 12 species using the same criteria. The program MEGA 6.6 (http://www.megasoftware.net/) [41] was used to align and construct an N-J tree with a bootstrap of 500. In the phylogenetic analysis, there was no BES1 protein found in algae but 6 were found in moss, indicating that BES1 may occur first in land plants. All of the proteins were classified into 5 groups according to the similarity of domain sequences. The BES1 genes were relatively conserved in the Amaranthaceae family; there are 6 and 7 BES1 proteins in spinach and sugar beet respectively, and 6 from each of them have counterparts. There was no correspondent for protein Bv4_085230 in spinach, which may have resulted from actual gene loss in spinach or imperfect annotation of the spinach genome. Interestingly, in groups A and C, the BES1 proteins from moss and spruce shared more similarity with one another than with fern, which was not consistent with plant classification rules (Fig 5).

### Useful tools for genome and genes

GBrowse, developed by the Generic Model Organism Database Project, is the most widely used genomic browser among organism databases, because the browser is both universal and customizable. It is possible to quickly and accurately locate regions of interest in spinach genes, and to display sequences and annotations visually. In SpinachDB, the GBrowse tool is highly configurable and portable; users can view eight data tracks including Gene, mRNA, CDS (Coding Sequence), SSR, SNP, 6-frame translation, the DNA/GC Content, and restriction sites of the spinach genome. The annotation and expression of genes that were measured using the FPKM method were integrated inside GBrowse, to provide users a way to comparably study gene function and expression in different samples. Furthermore, all of the useful SNP data are also available in GBrowse, allowing users to view the SNP information from cultivars of interest and referent species. This is robust tool for breeders to identify markers that may relate to traits of interest.

All sequences and annotations of spinach genome, genes, proteins and ESTs were deposited in SpinachDB. SpinachDB provides a BLAST search interface which allows users to perform sequence similarity searches against the spinach assembled genome, genes, cDNA, CDS, EST and proteins. Since the annotations using NR, KEGG, KOG, Swiss-Prot and TrEMBL databases were deposited in SpinachDB, users can also perform keyword searches in SpinachDB, which will obtain a list of annotated genes with the specific keyword. Similar searches could also be performed to search orthologs, paralogs and gene families.

## Supporting Information

S1 File Annotation, classification, and expression information of spinach genes in tables. (XLSX)
